# The Burden of Diabetic Gangrene: Prognostic Determinants of Limb Amputation from a Tertiary Center

**DOI:** 10.3390/medicina61101817

**Published:** 2025-10-11

**Authors:** Florin Bobirca, Dan Dumitrescu, Octavian Mihalache, Horia Doran, Cristina Alexandru, Petronel Mustatea, Liviu Mosoia-Plaviciosu, Anca Pantea Stoian, Vlad Padureanu, Anca Bobirca, Traian Patrascu

**Affiliations:** 1 Faculty of Medicine, Carol Davila University of Medicine and Pharmacy, 050474 Bucharest, Romania; 2 1st Surgery Department, Ion Cantacuzino Clinical Hospital, 011437 Bucharest, Romania; 3 Internal Medicine and Rheumatology Department, Ion Cantacuzino Clinical Hospital, 011437 Bucharest, Romania; 4 Surgery Department, Carol Davila Emergency Military Hospital, 010825 Bucharest, Romania; 5 Nicolae Paulescu National Institute of Diabetes, Nutrition and Metabolic Diseases, 030167 Bucharest, Romania; 6 Department of Internal Medicine, University of Medicine and Pharmacy Craiova, 200349 Craiova, Romania

**Keywords:** diabetic foot, limb amputation, gangrene, diabetes complications

## Abstract

*Background and Objectives:* Diabetic foot gangrene remains a major cause of lower limb amputation, driven by vascular, neuropathic, and infectious mechanisms. Identifying predictors for amputation type is essential to optimizing outcomes and reducing disability. We aimed to analyze the burden of diabetic foot gangrene and the patients’ characteristics according to the type of surgery, minor or major amputations. *Methods:* We conducted a retrospective observational study including 295 diabetic patients who underwent surgery for foot lesions at a Romanian tertiary center (January 2023–December 2024). Patients were classified according to surgical outcome as minor (toe/foot-level) or major (below/above-knee) amputations. Clinical, demographic, and pathological variables were compared between groups. Statistical analysis was performed with IBM SPSS Statistics 20.0. Categorical variables were expressed as frequencies and percentages, and continuous variables as mean ± SD or median (min–max). Group comparisons used Student’s *t*-test, Mann–Whitney U, Chi-square, or Fisher’s exact test, and binary logistic regression was applied to calculate odds ratios (OR) with 95% confidence intervals (CI). *Results:* Among the patients included (mean age 64.8 ± 10.8 years; 69.2% male), 191 (64.7%) underwent minor amputations/debridement and 104 (35.3%) required major amputations. Patients with major amputations were older (66.8 ± 11.3 vs. 63.7 ± 10.4 years, *p* = 0.012) and less frequently male (56.7% vs. 75.9%, *p* = 0.001). Lesion extension to the foot or beyond strongly predicted major amputation (*p* < 0.001). Peripheral arterial disease was more prevalent in the major group (85.6% vs. 65.4%, OR = 3.13, 95% CI = 1.68–5.84), while neuropathy was associated with minor procedures (12.6% vs. 3.8%, *p* = 0.015). Anemia (70.2% vs. 56.5%, *p* = 0.021) and leukocytosis (68.3% vs. 49.2%, *p* = 0.002) were also independent predictors of major amputation. *Conclusions:* The study highlights the need for early detection, coordinated multidisciplinary care, and personalized assessment of diabetes burden and its complications to minimize the risk of major limb amputation.

## 1. Introduction

Diabetes mellitus is one of the most pressing challenges for global healthcare systems. More than 537 million individuals are currently diagnosed, and an additional 240 million remain undiagnosed, generating annual healthcare costs exceeding 900 billion dollars [1]. Among its complications, the diabetic foot is particularly debilitating, frequently leading to hospitalizations, complex treatments, and limb amputations, with a profound impact on patients’ quality of life [1].

The diabetic foot results from the interplay of three major pathophysiological mechanisms: peripheral arterial disease, neuropathy, and infection [2,3]. Each mechanism contributes individually or in combination, but typically one predominates and dictates the clinical course. Wet gangrene is an infected necrotic lesion requiring emergency surgery; ischemic necrosis (dry gangrene) reflects impaired arterial supply, and neuropathic ulceration arises from diminished sensitivity and increased plantar pressure [2,3]. The severity and anatomical extension of these lesions directly influence prognosis and the level of amputation required.

Epidemiological data highlight the magnitude of the problem: the lifetime risk for a diabetic patient to develop a foot lesion ranges between 19 and 34%, and recurrence occurs in up to two-thirds of cases within five years [4,5]. Each year, more than 150,000 amputations are performed in the United States, a number that continues to rise [6]. In Europe, the prevalence of diabetic foot ulcers is reported at 5.1% among patients with diabetes [7,8], emphasizing the substantial health and economic burden of this condition.

Therapeutic advances, including wound care innovations [9,10,11,12], orthopedic management [13,14], and revascularization procedures [15,16], have improved local outcomes. Moreover, guidelines emphasize the role of multidisciplinary “diabetes teams” composed of surgeons, diabetologists, vascular surgeons, orthopedists, and infectious disease specialists [17,18]. Nevertheless, despite these improvements, the rate of major amputations remains high, particularly in patients with advanced age, cardiovascular disease, or chronic kidney disease [17,19].

Given these challenges, the identification of prognostic determinants that differentiate between minor and major amputations is of critical importance. Early recognition of such factors may facilitate personalized strategies, reduce disability, and improve survival.

The aim of this study is to analyze the burden of diabetic gangrene and to evaluate the clinical, demographic, and pathological characteristics of patients according to the type of surgery—minor or major amputations. Although therapeutic advances have improved the prognosis of patients with diabetic foot, the number of amputations remains high. This study is motivated by the need for a better understanding of the clinical characteristics and comorbidities that influence the type of surgery to allow for early and personalized interventions.

## 2. Materials and Methods

**Figure 1:**
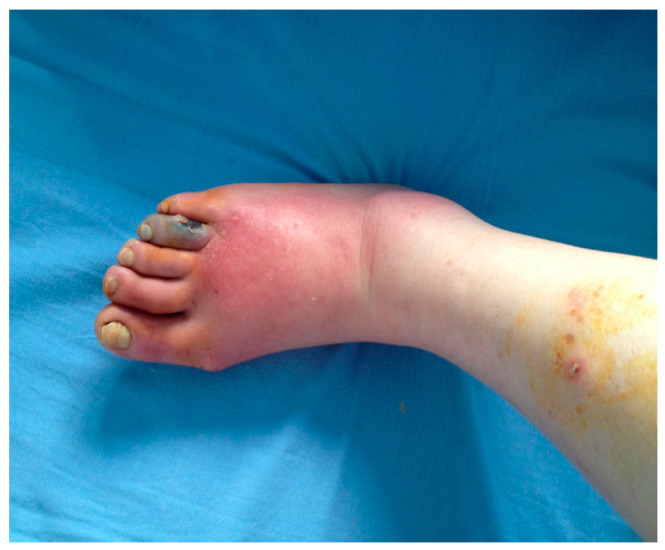
Wet gangrene of the toe; Dr Ion Cantacuzino Clinical Hospital Collection.

**Figure 2:**
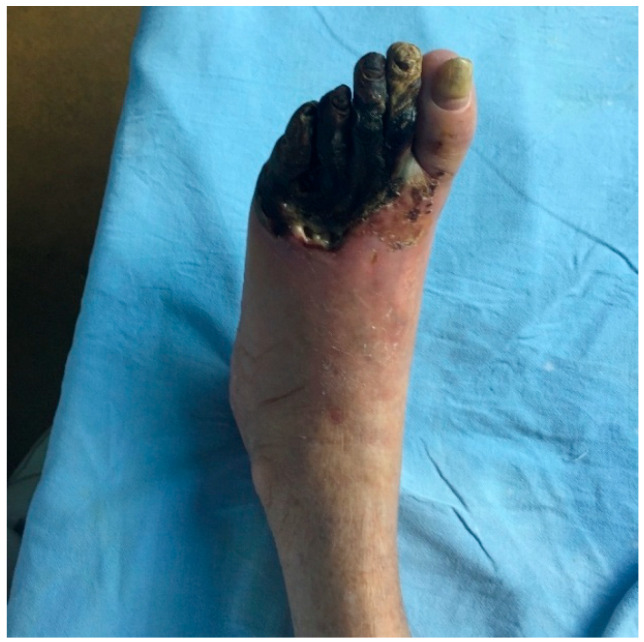
Ischemic necrosis (dry gangrene) of the forefoot; Dr Ion Cantacuzino Clinical Hospital Collection.

A retrospective, observational study was conducted in the Surgery Department of the I. Cantacuzino Clinical Hospital in Bucharest, between January 2023 and December 2024. The cohort consists of 295 patients with predominantly ischemic diabetic foot lesions who underwent surgery. The inclusion criteria were defined as patients ≥ 18 years with a confirmed diagnosis of diabetes mellitus and foot lesions requiring surgical intervention (debridement, minor or major amputation). The term “wet gangrene” is defined as necrotic tissue associated with clinical signs of infection (erythema, edema, purulent discharge) (Figure 1), whereas “ischemic necrosis” (dry gangrene) was defined as non-infected necrotic lesions in the context of documented peripheral arterial disease (Figure 2).

The classification of lesions was performed clinically by at least two independent senior surgeons, based on physical examination and standard diagnostic protocols. Neuropathy was diagnosed using clinical sensory testing (monofilament, vibration sense, thermal discrimination). Arteriopathy (peripheral arterial disease) was documented using the ankle-brachial index (ABI), oscillometry, and/or imaging when available. ABI was classified as mild (0–1.5), moderate (1.5–2.5), or severe (>2.5). Infection was established based on the IWGDF/IDSA criteria, requiring at least two local signs of inflammation (erythema, warmth, tenderness, swelling, purulent secretion).

Patients under the age of eighteen, diabetic foot lesions that needed just conservative treatment (without surgery), and those without diabetes were excluded.

For the purposes of analysis, the cohort was divided into two groups, minor and major amputation, according to the most extensive surgical procedure required. The type of surgery performed was transmetatarsal resection of toe/toes, transphalangeal resection, excisional debridement, below-knee amputation, and above-knee amputation. The first two are considered minor amputations (transmetatarsal resection and transphalangeal resection), whereas the last two are major amputations (below and above the knee), thus constituting 2 categories.

For the purpose of this study, a “favorable postoperative outcome” was defined as wound healing with the absence of local/systemic infection and patient survival until discharge.

Statistical Analysis

The descriptive and analytical statistics were conducted using IBM SPSS Statistics version 20.0. Categorical variables’ values were recorded as numbers and percentages, but continuous variables were reported as mean and standard deviation (SD), median, and minimum and maximum, depending on the normal distribution. To identify any significant associations, the data were examined using Student’s *t*-test and Mann–Whitney U test with a two-tailed hypothesis for continuous data, and Pearson Chi-square and Fisher’s exact test were used for categorical data. For OR and 95CI, binary logistic regression was used.

## 3. Results

A total of 295 patients with diabetic foot lesions requiring surgical intervention were included (Table 1). The mean age of the study population was 64.8 ± 10.8 years, with a predominance of male patients (69.2%, *n* = 204).

The vast majority of cases were diagnosed with type 2 diabetes mellitus (96.9%, *n* = 286), whereas only 3.1% (*n* = 9) presented type 1 diabetes.

Treatment distribution was relatively balanced, with 44.4% receiving insulin and 55.6% being treated with oral antidiabetic medication.

Regarding associated comorbidities, cardiovascular diseases were highly prevalent, affecting 70.8% (*n* = 209) of patients.

Among these, 43.4% (*n* = 128) had two or more cardiovascular conditions, and 27.5% (*n* = 81) had a single comorbidity.

Microangiopathic complications of diabetes were also common: nephropathy was present in 35.9% (*n* = 106) of patients, retinopathy in 21.7% (*n* = 64), and neuropathy in 9.5% (*n* = 28).

Peripheral arterial disease, identified in 72.5% (*n* = 214) of the cohort, represented the most frequent underlying pathological mechanism.

In terms of lesion type, wet gangrene accounted for 61.4% (*n* = 181) of cases, while ischemic necrosis (dry gangrene) was identified in 38.0% (*n* = 112). Neuropathic ulcers were rare, with only two cases (0.7%).

When stratified by surgical outcome (Table 2), 191 patients (64.7%) underwent minor amputations or debridement, whereas 104 patients (35.3%) required major amputations. Patients in the major amputation group were significantly older than those in the minor group (66.8 ± 11.3 vs. 63.7 ± 10.4 years, *p* = 0.012), suggesting that disease progression with age increases the likelihood of extensive surgery.

Sex distribution showed that male predominance was significantly higher in the minor group (75.9% vs. 56.7%, *p* = 0.001; OR = 2.40, 95% CI = 1.44–4.01), possibly reflecting differences in healthcare access or disease progression.

The extension of the primary lesion demonstrated a strong association with surgical outcome. In the minor amputation group, the majority of lesions were localized to the toes (59.2%), whereas in the major amputation group, lesions most frequently involved the foot (66.3%) or extended to the calf/thigh (29.9%).

This distribution was statistically significant (*p* < 0.001), highlighting lesion extent as a major determinant of amputation level.

By contrast, lesion type (wet vs. dry gangrene) did not differ significantly between groups (*p* = 0.550). Analysis of comorbidities indicated that peripheral arterial disease was substantially more frequent in patients undergoing major amputations (85.6% vs. 65.4%, *p* < 0.001; OR = 3.13, 95% CI = 1.68–5.84).

Peripheral neuropathy, however, was more prevalent in patients with minor amputations (12.6% vs. 3.8%, *p* = 0.015; OR = 0.28, 95% CI = 0.09–0.83).

Diabetic retinopathy showed a borderline association, which is more common in the major group (27.9% vs. 18.3%, *p* = 0.057).

These findings emphasize the complex role of vascular versus neuropathic complications in determining surgical outcomes.

Hematological abnormalities further distinguished the two subgroups.

Anemia was more frequently observed in patients with major amputations (70.2% vs. 56.5%, *p* = 0.021; OR = 1.81, 95% CI = 1.09–3.01), while leukocytosis was also significantly associated with extensive procedures (68.3% vs. 49.2%, *p* = 0.002; OR = 2.22, 95% CI = 1.35–3.66).

Additionally, a history of previous surgical intervention was more common in the major amputation group (47.1% vs. 28.8%, *p* = 0.002; OR = 2.20, 95% CI = 1.34–3.63), suggesting that recurrent infections or progression after prior procedures increase the risk of major amputation.

Hospitalization-related outcomes were comparable across groups. The average length of stay was 8.7 ± 4.8 days, with no significant differences between minor and major amputations (*p* = 0.394).

Postoperative antibiotic therapy was administered in almost all patients (98.6%), with a mean duration of 5.2 ± 3.1 days for the minor group and 4.8 ± 2.9 days for the major group (*p* = 0.614). Immediate postoperative outcomes were favorable in the vast majority, although mortality was higher among patients with major amputations (3.8% vs. 1.6%, *p* = 0.040).

Overall, the results confirm that older age, lesion extension, vascular pathology, hematological abnormalities, and prior surgical history were strongly associated with the likelihood of undergoing a major amputation.

These findings directly support the primary aim of the study, namely to identify clinical and pathological factors that determine the type of surgical intervention in diabetic gangrene.

## 4. Discussion

Recent IWGDF/IDSA 2023 guidelines [18] reinforce the necessity of multidisciplinary care, recommending that management should involve vascular surgeons, diabetologists, infectious disease specialists, and wound care experts. Our findings support this approach, as patients with ischemia and infection required more extensive interventions, highlighting the need for coordinated decision-making. Also, a study conducted in a hospital in Spain stipulated that after the introduction of a specialized team, the rate of major amputations decreased by 66%. (*p* < 0.001) [19].

In this study, we obtained a mean age of 64.8 years, which can be compared with a Chinese study [20] conducted on a group of 992 patients in which the mean age was 65.1 years. In addition, this study also highlighted a higher mean age in the case of those who had major amputations compared to minor ones (69.4 vs. 62.9, *p* = 0.146), which was also confirmed in our article. (66.8 vs. 63.7, *p* = 0.012).

The average age and gender of the patients are other factors that were analyzed, thus demonstrating that patients over 60 years and men are more susceptible to undergoing an amputation. Another lecture confirms the values obtained by this study, this time with a group of 13,774 patients, highlighting the fact that the average age of the patients was 72.4 years and the male gender represented 63.1% of the patients [21]. Also, another study conducted in the USA on a sample of 6839 patients who underwent major amputations revealed a predominance of patients over 60 years (67%, *n* = 4609) and male patients (62%, *n* = 4182) [22].

Regarding primary lesions, although in our study the most common lesion was wet gangrene (61.4%, *n* = 181), another study from Australia revealed that neuropathic ulceration was the predominant lesion (85%, *n* = 63), thus marking a major difference from our results [23].

Another aspect addressed in this study is diabetes complications that are closely related to microangiopathy and the impairment of the cardiovascular, renal, and nervous systems. From the literature, it is noteworthy to mention that a study on patients with diabetic foot pathology and associated peripheral arterial disease, surgical interventions such as major amputations amounted to 33.8%, which is comparable to the values obtained in our study in the case of above- and below-knee amputations (35.3%, *n* = 104) [24]. Likewise, another study that quantified peripheral arterial disease in patients with diabetes revealed that 56% (*n* = 141) of patients who had minor amputations suffered from arteriopathy (peripheral arterial disease), while in our study, the percentage was 65.4 (*n* = 125) [25].

Another complication of diabetes mellitus, diabetic retinopathy, was observed in our study in 27.9% of cases (*n* = 29) with major amputations and in 18.3% (*n* = 35) of patients with minor amputations, which is different from what was published by Gong et al. who obtained 35.2% (*n* = 6) in those with major amputations and 35.3% in those with minor amputations (*n* = 20) [20].

Laboratory analyses highlight changes that are especially noticeable in the case of major amputations. In our study, we highlighted anemia in 70.2% (*n* = 73) of patients and renal impairment in 37.5% (*n* = 39) of patients. Costa et al. [26] noted in their study that patients with major amputations had anemia in 89.6% cases. Kharahan et al. noted that 25.7% of patients with major amputations had chronic kidney disease [27]. Also, in other studies, chronic kidney disease is an independent risk factor for plantar ulcers and major lower limb amputation, independent of peripheral arterial disease [28,29].

In our study, the length of hospital stay was similar between the two groups (minor and major amputation), with a range of 8.8 ± 4.6/8.6 ± 5.1, and it was also similar to the results of other specialized studies. The average length of hospital stay for patients admitted for problems related to diabetic foot varies significantly between clinical details and geographical locations, usually between 8 and 18 days. The impact of systemic toxicity and infection severity on hospitalization duration was highlighted by Wukich et al., who observed a median length of stay of 8 days for patients with severe diabetic foot infections in a U.S. academic facility, compared to 5 days for those with mild infections [30]. Similar patterns were seen in a Turkish cohort, where higher Wagner grades and raised inflammatory markers (CRP, WBC) were associated with longer length of stay, whereas an inverse relationship with amputation rate indicated that some patients had surgery earlier [31]. According to a Chinese single-center study, patients who needed amputations were admitted for a considerably longer period of time (mean 32.8 vs. 16.5 days), and their length of stay increased gradually, reaching a median of 31 days [32].

Prior minor amputations or surgical debridements are linked to increased reamputation rates or the path to major amputation, according to certain studies, particularly in patients with multiple risk factors [33]. A poor healing reaction frequently signifies an imbalance or a malfunction in the treatment of diabetes rather than a surgical failure [34].

The association between gangrene and risk of amputation is further highlighted by a large meta-analysis by Sen *p* et al. [35,36], which identified gangrene as one of the strongest predictors among diabetic foot ulcer patients, osteomyelitis, ulcer history, male sex, smoking, lower BMI, and elevated white blood cell count. This underscores how the presence of sepsis accelerates the path to limb amputation.

Long-term diabetes (≥15 years), poor glycemic control, insulin therapy, hypertension, heart disease, renal impairment, stroke, and the presence of gangrene and ischemia are other independent predictors of major lower limb amputation, according to a recent retrospective study conducted in Jordan involving patients with type 2 diabetes associated with diabetic foot syndrome [37,38].

Limitations

This study has a number of limitations that should be noted. The first is related to the laboratory aspect; no data are available regarding the levels of glycated hemoglobin, procalcitonin, or reactive protein C. Microbiological data were partially collected and will be the subject of further research, with the goal of collecting a larger number of patients with valid bacteriological examinations. Although it is a representative Romanian center, the data reflects the area standard in many aspects.

The study did not include mid- and long-term follow-up of patients, which limits the interpretation of clinical evolution after discharge. Future investigations, with assessments at 6–12 months or even longer, are needed to provide a complete picture of the impact of the interventions and the prognostic factors identified, and will be included in other studies.

Perspectives

Multicenter cohort studies with standardized medical classification methods should be the main focus of future research. Eventually, the application of AI and machine learning algorithms may improve predictive accuracy and assist in identifying patients who need aggressive intervention or early angioplasty. Additionally, community-based screening technologies and telemedicine systems may decrease late-stage diagnosis. The adoption of international standards, such as the IWGDF 2023 recommendations, could harmonize data collection and clinical practice, enabling comparability across centers.

## 5. Conclusions

This study highlights that, although most patients with diabetic foot had an immediate favorable postoperative outcome, there is a clinical profile associated with an increased risk of major amputation. Thus, older patients with extension of lesions to the foot or lower limb, with documented peripheral arterial disease, anemia, leukocytosis, and surgical history, were most likely to require extensive amputations. These results emphasize the need for early identification of risk factors and the implementation of a multidisciplinary approach to limit progression to major amputations and improve the quality of life of patients with diabetes.

In the delicate and complex context of diabetic foot pathology, this study emphasizes the high occurrence of limb amputation, especially major amputation.

Prevention, education, and prompt intervention are crucial because these findings highlight the significance of early detection and management of ischemic lesions associated with infection, ideally through collaborative teams of specialists and organized diabetic foot programs.

## Figures and Tables

**Table 1:** Characteristics of the study group.

Patients Characteristics	Total N = 295
Age	64.8 ± 10.8
Male Sex	204, 69.2%
Type of diabetes	
Type 1	9, 3.1%
Type 2	286, 96.9%
Diabetes treatment	
1 (Insulin)	131, 44.4%
2 (Oral antidiabetics)	164, 55.6%
Extension of the primary lesion	
Toe/Toes	120, 40.7%
Foot	129, 43.7%
Below-knee (Calf)/Above-knee (Thigh)/extensive	46, 15.6%
Type of lesion Wet gangrene (septic risk)—1 Ischemic necrosis/dry gangrene (peripheral arteriopathy)—2 Diabetic foot ulcer (Diabetic neuropathy)—3	181, 61.4% 112, 38.0% 2, 0.7%
Cardiovascular (CV) comorbidities	
Without comorbidities	86, 29.2%
One CV comorbidity	81, 27.5%
2 or more CV comorbidities	128, 43.4%
Chronic kidney disease	106, 35.9%
Diabetic retinopathy	64, 21.7%
Neuropathy	28, 9.5%
Arteriopathy	214, 72.5%
Neuropathy and Arteriopathy	53, 18.0%
Ankle-Brachial Index/Oscillometry N = 236	
Mild (0–1.5)	187, 79.2%
Moderate (1.5–2.5)	22, 9.3%
Severe (>2.5)	27, 11.4%
Type of surgery:	
Transmetatarsal resection of toe/s	124, 42.0%
Transphalangeal resection	15, 5.1%
Excisional debridement	52, 17.6%
Below-knee amputation	51, 17.3%
Above-knee amputation	53, 18.0%
Previous surgical intervention	104, 35.3%
Anemia	181, 61.4%
Leukocytosis	165, 55.9%
Blood sugar upon admission	212.9 ± 92.6
Duration of hospitalization (mean ± SD)	8.7 ± 4.8
Postoperative antibiotherapy	291, 98.6%
Length of antibiotherapy (days), mean ± SD	5.2 ± 3.1
Immediate postoperative outcome	
Favorable	267, 90.5%
Slow favorable	13, 4.4%
Major amputation	8, 2.7%
Death	7, 2.4%

**Table 2:** Patients’ characteristics according to the type of surgery.

Patients Characteristics	Minor Amputation N = 191, 64.7%	Major Amputation N = 104, 35.2%	*p*-Value	OR (95% CI)
Age	63.7 ± 10.4	66.8 ± 11.3	0.012	1.027 (1.004–1.051)
Male Sex	145, 75.9%	59, 56.7%	0.001	2.404 (1.443–4.005)
Type of diabetes			0.903	1.090 (0.260–4.450)
Type 1	6, 3.1%	3, 2.9%		
Type 2	185, 96.9%	101, 97.1%		
Diabetes treatment			0.656	1.070 (0.780–1.460)
1 (Insulin)	83, 43.5%	48, 46.2%		
2 (Oral antidiabetics)	108, 56.5%	56, 53.8%		
Extension of the primary lesion			<0.001	1.353 (0.686–2.719)
Toe/s	113, 59.2%	7, 6.7%		
Foot	60, 31.4%	69, 66.3%		
Below-knee/Above-knee/Extensive	18, 9.4%	28, 29.9%		
Type of lesion			0.550	
Wet gangrene (septic risk)—1	118, 61.8%	63, 60.6%		
Ischemic necrosis/dry gangrene (peripheral arteriopathy)—2	71, 37.2%	41, 39.4%		
Diabetic foot ulcer (Diabetic neuropathy)—3	2, 1.0%	0		
Cardiovascular (CV) comorbidities			0.203	0.820 (0.424–1.580)
Without	61, 31.9%	25, 24.0%		
One CV comorbidity	54, 28.3%	27, 26.0%		
2 or more CV comorbidities	76 39.8%	52, 50.0%		
Chronic kidney disease	67, 35.1%	39, 37.5%	0.679	1.110 (0.676–1.823)
Diabetic Retinopathy	35, 18.3%	29, 27.9%	0.057	1.723 (0.981–3.029)
Neuropathy	24, 12.6%	4, 3.8%	0.015	0.278 (0.094–0.825)
Arteriopathy	125, 65.4%	89, 85.6%	<0.001	3.133 (1.680–5.841)
Neuropathy and Arteriopathy	42, 22.0%	11, 10.6%	0.015	
Ankle-Brachial Index/Oscillometry N = 236			0.370	
Mild (0–1.5)—1	128, 77.1%	59, 84.3%		
Moderate (1.5–2.5)—2	16, 9.6%	6, 8.6%		
Severe (>2.5)—3	22, 13.3%	5, 7.1%		
Previous surgical intervention	55, 28.8%	49, 47.1%	0.002	2.203 (1.341–3.629)
Anemia	108, 56.5%	73, 70.2%	0.021	1.810 (1.089–3.009)
Leucocitosis	94, 49.2%	71, 68.3%	0.002	2.220 (1.345–3.664)
Blood sugar upon admission	216.4 ± 98.4	206.5 ± 81.2	0.544	0.999(0.996–1.001)
Type of surgery:			<0.001	
Transmetatarsal resection of the toe/s	124, 64.9%	0		
Transphalangeal resection	15, 7.9%	0		
Excisional debridement	52, 27.2%	0		
Below-knee amputation	0	51, 49.0%		
Above knee-amputation	0	53, 51.0%		
Duration of hospitalization (mean ± SD)	8.8 ± 4.6	8.6 ± 5.1	0.394	
Postoperative antibiotherapy	187, 97.9%	104, 100%	0.301	
Length of antibiotherapy (days), mean ± SD	5.31 ± 3.2	4.8 ± 2.9	0.614	
Immediate postoperative outcome			0.040	
Favorable	169, 88.5%	98, 94.2%		
Slow favorable	11, 5.8%	2, 1.9%		
Major amputation	8, 4.2%	0		
Death	3, 1.6%	4, 3.8%		

## Data Availability

The original contributions presented in this study are included in the article. Further inquiries can be directed to the corresponding author(s).
